# Strain-Dependent Activity of Zika Virus and Exposure History in Serological Diagnostics

**DOI:** 10.3390/tropicalmed5010038

**Published:** 2020-03-03

**Authors:** Kelli L. Barr, Erika R. Schwarz, Dhani Prakoso, Kehkashan Imtiaz, Ruiyu Pu, J. Glenn Morris, Erum Khan, Maureen T. Long

**Affiliations:** 1 Department of Biology, Baylor University, Waco, TX 76798, USA; 2 Department of Comparative Diagnostic and Population Medicine, College of Veterinary Medicine, University of Florida, Gainesville, FL 32608, USA; eschwarz@ufl.edu (E.R.S.); dprakoso@ufl.edu (D.P.); pur@ufl.edu (R.P.); longm@ufl.edu (M.T.L.); 3 Department of Pathology and Laboratory Medicine, Aga Khan University, Karachi 74800, Pakistan; kehkashan.imtiaz@aku.edu (K.I.); erum.khan@aku.edu (E.K.); 4 Emerging Pathogens Institute, University of Florida, Gainesville, FL 32601, USA; JGMorris@epi.ufl.edu

**Keywords:** Zika virus, flavivirus, cross-reactivity, neutralization, diagnostics, serology, plaque reduction neutralization test, flavivirus exposure

## Abstract

Zika virus (ZIKV) circulates as two separate lineages, with significant genetic variability between strains. Strain-dependent activity has been reported for dengue virus, herpes simplex virus and influenza. Strain-dependent activity of subject specimens to a virus could be an impediment to serological diagnosis and vaccine development. In order to determine whether ZIKV exhibits strain-dependent activity when exposed to antibodies, we measured the neutralizing properties of polyclonal serum and three monoclonal antibodies (ZKA185, 753(3)C10, and 4G2) against three strains of ZIKV (MR−766, PRVABC59, and R103454). Here, MR−766 was inhibited almost 60% less by ZKA185 than PRVABC59 and R103454 (*p* = 0.008). ZKA185 enhanced dengue 4 infection up to 50% (*p* = 0.0058). PRVABC59 was not inhibited by mAb 753(3)C10 while MR−766 and R103453 were inhibited up to 90% (*p* = 0.04 and 0.036, respectively). Patient serum, regardless of exposure history, neutralized MR−766 ~30%−40% better than PRVABC56 or R103454 (*p* = 0.005−0.00007). The most troubling finding was the significant neutralization of MR−766 by patients with no ZIKV exposure. We also evaluated ZIKV antibody cross reactivity with various flaviviruses and found that more patients developed cross-reactive antibodies to Japanese encephalitis virus than the dengue viruses. The data here show that serological diagnosis of ZIKV is complicated and that qualitative neutralization assays cannot discriminate between flaviviruses.

## 1. Introduction

Shortly after the identification of Zika virus (ZIKV) as the causative agent of microcephaly, researchers had released reagents and assays to assist in research and diagnostics [1,2]. ZIKV can be detected by RT-PCR in urine for 10–14 days after symptom onset but only for approximately 3 days from serum [3,4,5,6]. Unfortunately, urine testing or paired testing of serum and urine is rarely performed on subjects presenting to health care with a dengue-like illness. For most subjects with ZIKV or other flaviviral infection, the short duration of detectable viremia leads to the reliance on serological testing [3,7]. When undetected by RT-PCR, diagnosis is made following guidelines that require the presence of both virus-specific IgM antibodies and virus-specific neutralizing antibodies [7,8]. While clinicians use IgM ELISA to screen for infection, the plaque reduction neutralization test (PRNT) with ZIKV, dengue virus (DENV) and other endemic flaviviruses is recommended by the Centers for Disease Control and Prevention ( CDC) to confirm diagnosis when RT-PCR is negative, and IgM ELISA is “not negative” [7]. Unfortunately, even PRNT can be problematic for diagnosis with over half of patients exhibiting significant neutralization of ZIKV, DENV, and other flaviviruses when positive for ZIKV IgM [9].

ZIKV co-circulates with other flaviviruses, including DENV, West Nile virus (WNV), Japanese Encephalitis virus (JEV) and Yellow Fever virus (YFV). Antibody-based assays can be problematic as serological cross-reactivity can confound results [10,11,12]. Furthermore, previous exposures and co-infections can further complicate diagnostic tests [13,14,15]. Additional difficulties arise when diagnosing clinical samples in different locations as reference virus and antigen sources can produce divergent results in response to locally circulating viral isolates [16].

Prior to the 2016 outbreak, there were very few ZIKV isolates available for use, which included the prototype MR−766 isolate as well as IbH 30656 a Nigerian isolate and DAK AR 41524 from Senegal (BEIResources.org). MR−766 had been extensively characterized prior to the outbreak and was the reference strain used by many researchers in the early days following emergence [6,17,18,19,20]. Within a year of emergence, the CDC prototype PRVABC59 was released for use through BEI resources and many labs began using this modern isolate for their research and diagnostics as it was readily available and had been genetically characterized [21]. However, MR−766 continues to be widely used for the characterization of mAbs [22,23,24]. MR−766 was also used to develop ZIKV diagnostic primer and probe sets which comprise the CDC Zika real-time PCR and the CDC Trioplex real-time PCR assay and are widely used [6,25,26,27].

Strain-specific antigenic variability has been described for flaviviruses [28,29,30]. Studies characterizing ZIKV mAbs have documented strain-specific neutralization of ZIKV isolates, which suggests that ZIKV epitopes are as varied as the other flaviviruses [31]. We hypothesized that serological testing of clinical specimens would reflect the ZIKV strain used and that patient exposure history could confound results. The data herein illustrate that the magnitude of ZIKV neutralization by polyclonal serum or mAbs is dependent upon ZIKV strain. The data also illustrate that ZIKV neutralization by patient serum may not indicate ZIKV exposure.

## 2. Results

### 2.1. RT-PCR Sensitivity is Strain Dependent

When equal quantities of infectious particles were amplified via RT-PCR using CDC diagnostic primers and probes, cycle threshold (Ct) values varied significantly between ZIKV strains (Table 1). Ct values for PRVABC59 were consistently higher than the other isolates, differing by as many as 5 cycles translating to a 0.08 fold reduction in detection as determined by the ΔΔCt method which further translated to a 3.57 log reduction in detection (*p*-value 0.0000014) (Table 1). Conversely, R103451 produced Ct values comparable to MR−766. However, pairwise comparisons indicated that R013451 was detected at a significantly lower average Ct than MR−766 (*p*-value 0.0035) (Table 1). R103451 was detected by a 1.5-fold increase over MR−766 translating to an increase of just 0.58 logs more detection. This may be a result of infectious particle:genome copy ratios, as both R103451 and PRVABC59 have genetically identical target sequences.

### 2.2. Monoclonal Antibody Neutralization of ZIKV and DENV is Strain/Serotype Dependent

**Figure 1:**
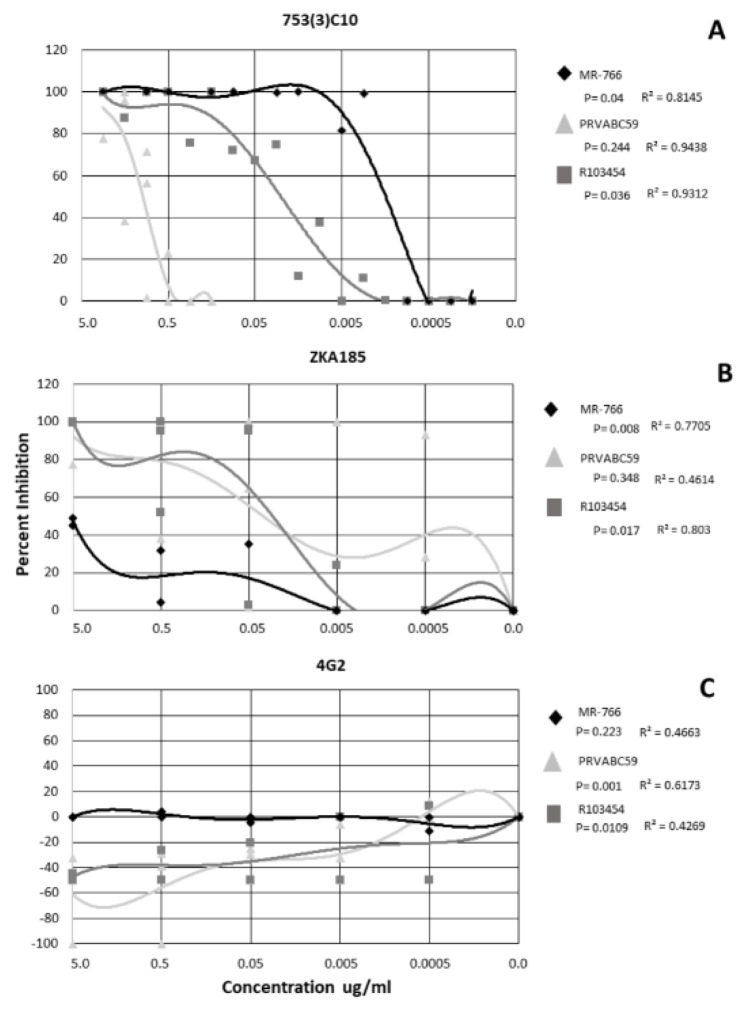
Neutralization and enhancement of Zika by 3 monoclonal antibodies. Non-linear regression was performed to identify significance. Each shape represents the average inhibition of 2 replicates.

Inhibitory concentrations were calculated for each dilution of the inhibition assays using the virus only control as the reference sample. Non-linear regression analysis was performed, and data interpolated using a polynomial curve. The mAb 753(3) C10 neutralized all ZIKV strains in a dose-dependent manner (r = 0.8145−0.9438) (Figure 1). MR−766 and R103453 were inhibited significantly more than PRVABC59 (*p* = 0.04 and 0.036, respectively) (Figure 1A). mAb 753(3) C10 inhibited ZIKV MR−766 at ~90% at 0.005 ug/ml, R103454 at ~70% at 0.05ug/ml and PRVABC59 was neutralized only at 5.0 ug/ml (Figure 1A).

ZKA185 inhibited ZIKV, on average, less than mAb 753(3) C10. MR−766 and R103454 were inhibited in a dose-dependent manner (r = 0.7705 and 0.803 respectively) (Figure 1B). MR−766 was inhibited significantly less than either PRVABC59 or R103454 (*p* = 0.008) (Figure 1B). R013454 was inhibited significantly more than either PRVABC59 or MR−766 (*p* = 0.017) (Figure 1B).

When treated with 4G2 antibody, PRVABC59 and R103454 were significantly enhanced (*p*= 0.001 and 0.0109, respectively). R values for all strains (0.4269−0.6173) did not indicate a dose-dependent response (Figure 1C). However, enhancement of PRVABC59 and R103454 occurred at mAb concentrations up to 0.005 ug/ml (Figure 1C). MR−766 was completely neutralized at all concentrations of 4G2 (Figure 1C).

**Figure 2:**
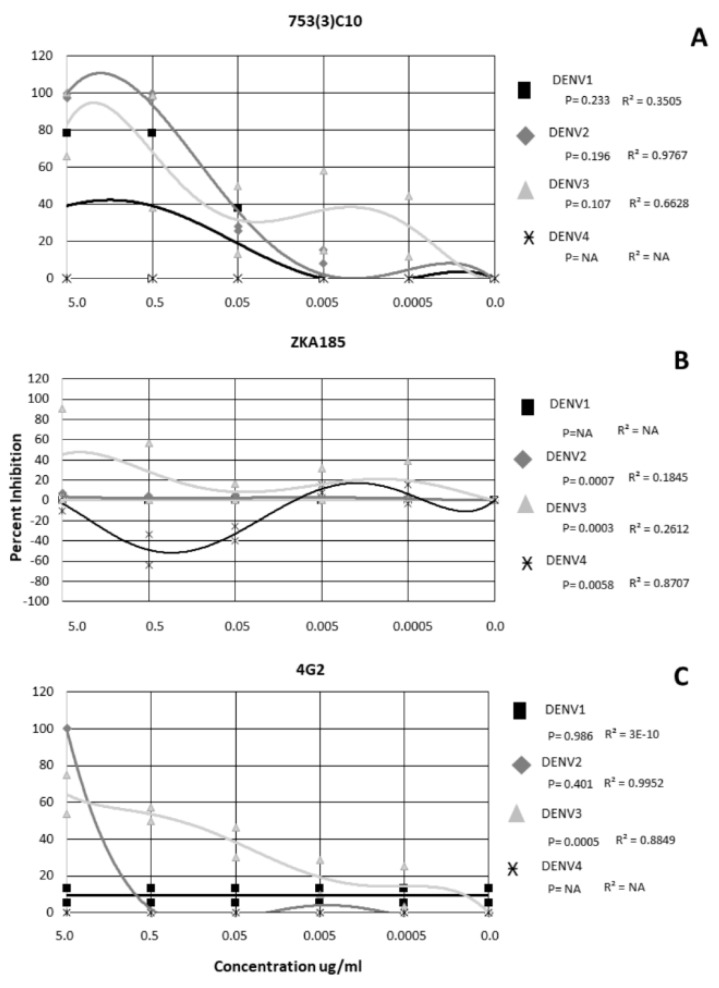
Neutralization and Enhancement of Dengue viruses by 3 monoclonal antibodies. Non-linear regression was performed to identify significance. Each shape represents the average inhibition of 2 replicates.

For mAb 753(3) C10, DENV2 and DENV3 were inhibited in a dose-dependent manner (r = 0.9767 and 0.6628, respectively) (Figure 2A). DENV4 was not neutralized at any concentration of 753(3) C10 (Figure 2A).

DENV 1−4 exhibited a serotype-specific response to mAb ZKA185 (Figure 2B). ZKA185 did not significantly neutralize DENV1 or DENV2 (Figure 2B). DENV3 was significantly more inhibited than DENV4 with viral inhibition occurring a 5.0 ug/ml, and 0.5 ug/ml (Figure 2B) (*p* = 0.003). DENV4 was significantly enhanced in the presence of ZKA184 (*p* = 0.0058) with enhancement occurring in a dose-dependent manner at both 0.5 ug/ml and 0.05 ug/ml (r = 0.8707) (Figure 2B).

The 4G2 antibody did not significantly inhibit DENV1 or DENV4 (Figure 2C). DENV2 was inhibited 100% at 5 µg/µL but not at any other concentration and wasn’t significant from the other serotypes (Figure 2C) (*p* = 0.401). DENV3 was neutralized in a dose-dependent manner (r = 0.8849) with ~62% inhibition at 5 µg/µL, 54% at 0.5 ug/mL, 39% at 0.05 µg/µL, 20% at 0.005 µg/µL and 12% at 0.0005 µg/µL (Figure 2C). Overall, DENV3 was inhibited significantly more than the other serotypes (*p* = 0.0005) (Figure 2C).

### 2.3. Subjects with Confirmed ZIKV Exposure with Unknown Flaviviral Exposure History Exhibit Strain-Specific Neutralization of ZIKV

**Figure 3:**
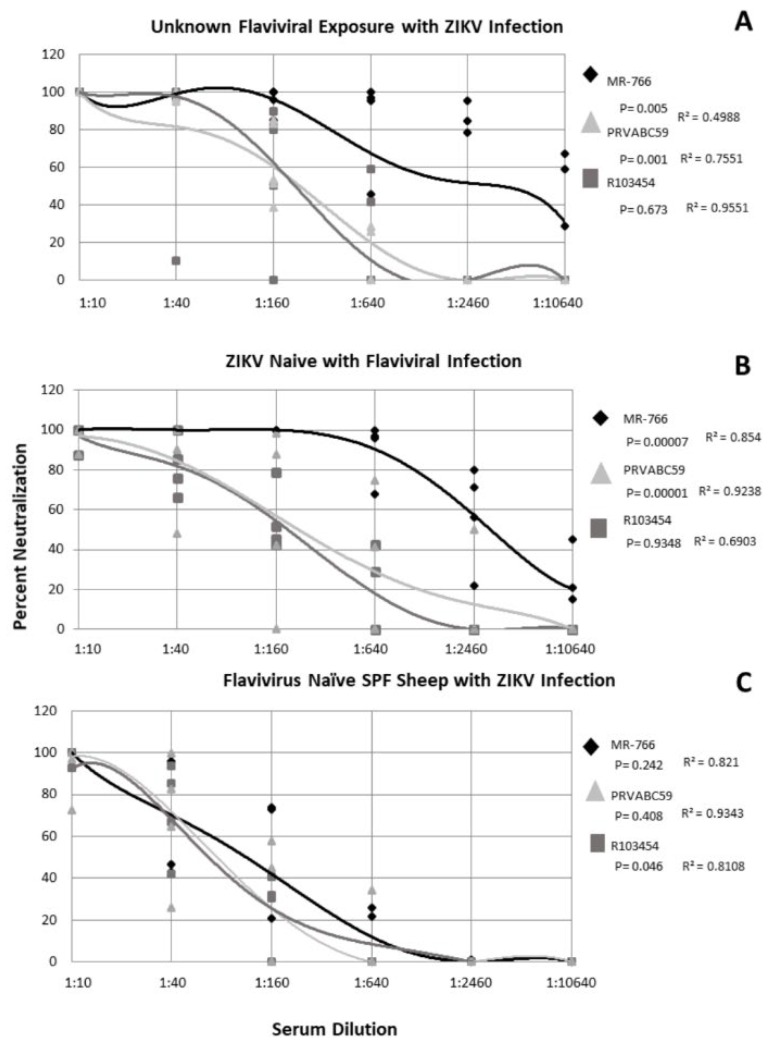
Neutralization of Zika viruses by subject serum with and without ZIKV exposure. Non-linear regression was performed to identify significance. Each shape represents the average inhibition of 2 replicates.

Five subjects with unknown exposure history with ZIKV infection were evaluated for their ability to neutralize multiple, geographically distinct isolates of ZIKV. The profile of neutralization was subject-specific though the data described here represent the non-linear regression derived from the combined response of all subjects in the group. The data indicate that ZIKV MR−766 was neutralized best by all subjects with significantly greater neutralization present over all other ZIKV strains at all serum dilutions (*p* = 0.005) (Figure 3A). An average neutralization of 80% was observed for MR−766 at the 1:640 serum dilution which indicates a robust immune response (Figure 3A). ZIKV PRVABC56 was neutralized significantly less than MR−766 by all subjects with neutralization dropping below 80% at the 1:160 serum dilution (*p* = 0.001) (Figure 3A). Unlike MR−766, PRVABC59 was neutralized in a dose-dependent manner (r = 0.7551), whereas MR−766 neutralization was not associated with a specific serum concentration (r = 0.4988) (Figure 3A). Subject neutralization of R103454 was dose dependent (r = 0.9551) but percent neutralization varied significantly between the subjects (*p* = 0.673) (Figure 3A).

### 2.4. ZIKV-Naïve Subjects with Flaviviral Exposure History Neutralize ZIKV in a Strain Dependent Manner

For all ZIKV-naïve subjects with a history of flaviviral exposure, ZIKV strains were neutralized in a dose-dependent manner (r = 0.6903−0.9238) (Figure 3B). ZIKV MR−766 was neutralized significantly more than either PRVABC59 or R013451 (*p* = 0.00007) (Figure 3B). Over 80% neutralization of all ZIKV strains was observed up to the 1:40 dilution with MR−766 neutralizing at least 80% out to the 1:640 dilution (Figure 3B). Even at the 1:10240 dilution, MR−766 was neutralized at an average of 20% and nearly 60% at the 1:2560 dilution (Figure 3B). ZIKV PRVABC59 and R103451 exhibited approximately 80% neutralization at the 1:40 serum dilution and at least 50% neutralization at the 1:160 serum dilution (Figure 3B). These elevated neutralization profiles suggested potential ZIKV circulation and exposure and thus, all subject specimens from this study were tested via RT-PCR, using the above primers. All subjects (n = 991) were negative for ZIKV nucleic acids (data not shown).

### 2.5. Flavivirus Naïve SPF Sheep with ZIKV Only Exposure Exhibit Similar Neutralization of ZIKV Strains

Because human exposure to flaviviruses is ubiquitous in all continents save Antarctica, we utilized specific pathogen free (SPF) sheep with a confirmed flavivirus naïve antigenic background to observe how ZIKV was neutralized in the absence of cross-reactive antibodies. Four SPF female sheep, naïve for flaviviral exposure, were infected with ZIKV R013451 and serum collected 4 weeks post infection. The data show that all ZIKV strains were neutralized in a dose-dependent manner (r = 0.8108−0.9343) (Figure 3C). R103454 was neutralized significantly more than either PRVABC59 or MR−766 (*p* = 0.046). This is likely an artifact of the initial exposure to R103454. Overall, this group exhibited a less robust neutralization compared with the other two exposure groups. Neutralization stopped at the 1:640 serum dilution for all subjects to all ZIKV strains (Figure 3C) while MR−766 was neutralized at 100% at this dilution for the either two exposure groups (Figure 3A,B) and PRVABC59 was neutralized up to 80% at this dilution (Figure 3A,B). This phenomenon is likely due to collection of serum being performed 4 weeks post infection prior to the complete rise in neutralizing antibodies.

### 2.6. Subjects with Confirmed ZIKV Exposure Exhibit Cross-Neutralization of Other Flaviviruses

For subjects with ZIKV infection but unknown flavivirus exposure, cross neutralization of other flaviviruses occurred for three out of five subjects at the 1:10 serum dilution (Table 2). Subject 50620 exhibited cross neutralization to DENV3 and JEV (Table 2). Subject 50622 exhibited cross-neutralization of DENV3, JEV, WNV, and YFV (Table 2). Subject 88 possessed neutralizing antibodies for DENV2, DENV3, and YFV (Table 2). Flaviviral naive subjects with ZIKV infection also neutralized other flaviviruses at the 1:10 dilution including DENV2, JEV, and YFV 4 weeks following experimental inoculation with ZIKV R013451 (Table 2).

### 2.7. ZIKV-Naïve Subjects with Previous Flavivirus Exposure Effectively Neutralize ZIKV and Other Flaviviruses

For all ZIKV-naïve subjects with flaviviral exposure history, neutralization of multiple flaviviruses was observed at the 1:10 dilution. Subject K−252 and K−200 completely neutralized all DENV serotypes as well as ZIKV while subjects K−315 and K−070 neutralized just 2 DENV serotypes (Table 2). Even though YFV does not circulate in Pakistan, subjects K−315 and K−070 neutralized YFV 87% and 97% at the 1:10 serum dilution, respectively (Table 2). Subject K−315 also neutralized WNV 85% and JEV 95% at the 1:10 serum dilution (Table 2). Subject K−070, K−200 and K−252 did not significantly neutralize WNV but subject K−200 significantly neutralized JEV (Table 2).

### 2.8. MR−766 Is Neutralized More Than DENV by ZIKV-Naïve Patient Serum

**Figure 4:**
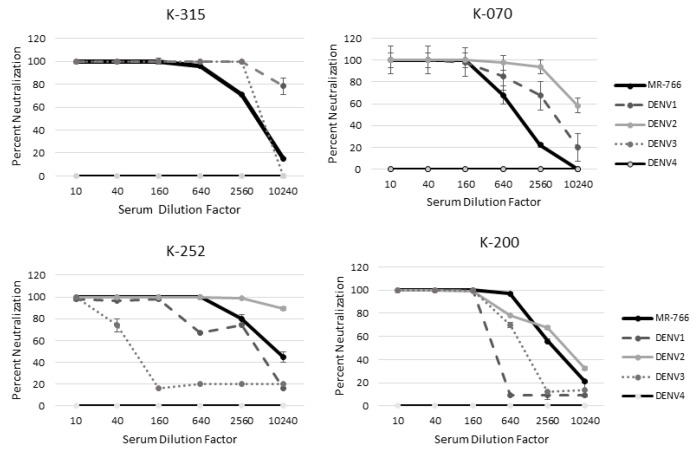
MR-766 is neutralized better than DENV in patients naïve for ZIKV. A quantitative PRNT was performed using serum from patients naïve for ZIKV exposure but with likely flavivirus exposure.

Quantitative PRNTs were performed for these subjects to determine whether infectious status could be determined. For these subjects, MR−766 was neutralized more than at least 1 DENV serotype out to the lowest serum dilution of 1: 10,240 (Figure 4). MR−766 was neutralized better than DENV 2 and 4 for all serum dilutions of patient K−315 (Figure 4). Patient K−070 neutralized MR−766 better than DENV 3 and 4 (Figure 4). Patient K−200 neutralized MR−766 better than all four DENV serotypes. Patient K−252 also neutralized ZIKV MR−766 better than DENV 1, 3, and 4 (Figure 4). All patients neutralized MR−766 better than DENV4 (Figure 4).

## 3. Discussion

The use of monoclonal antibodies for ZIKV therapeutics and vaccine development is a prominent focus of ZIKV research, with most neutralizing mAbs mapped to the fusion loop on the envelope protein [32]. The monoclonal antibodies used in this study neutralized and/or enhanced ZIKV and DENV in a strain-dependent manner. Similar results have been reported for other mAbs which has been a complicating factor for developing useful therapeutics [33]. Many commercially available ZIKV mAbs have been isolated from human donors in regions where multiple flavivirus co-circulate and for which there is no available exposure history [33,34]. It is of interest to note that the ZIKV isolates in this study displayed much higher sensitivity to mAb 753(3) C10 (a DENV mAb) than DENV which may indicate that the source donor had a prior exposure to ZIKV [35].

An interesting feature of these mAbs was their enhancement of ZIKV and DENV. The 4G2 antibody is a widely used flaviviral antibody that was derived from DENV2 and shown to react with all DENV serotypes and a variety of flaviviruses including ZIKV [36]. The data show that, like Henchal et al., 4G2 reacted with all four serotypes of DENV and ZIKV [36]. While 4G2 neutralized ZIKV MR−766, it significantly enhanced PRVABC59 at multiple concentrations. Antibody dependent enhancement is a common feature of DENV by which non-neutralizing or weakly neutralizing antibodies bind to the infecting strain. The antibody: virus complex is then recruited to macrophage cells where it replicates at increased levels leading to severe infection [37].

A second mechanism for antibody enhancement is through intracellular processes by the suppression of innate cellular immunity allowing for increased spread of infection [37]. Since macrophages or other monocytes were not used in this study, the enhancement we observed was likely due to antibody mediated enhancement (AME). Like ADE, AME occurs when antibodies bind to virus particles forming complexes. These complexes interact with cell surface receptors and promote entry into host cells leading to increased levels of viral replication via suppression of innate immune processes and inflammatory cascades [38,39]. While this process is associated with Fc receptor-bearing monocytes, it is also possible for these virus–antibody complexes to infect other cell types and suppress innate immunity.

ZIKV is described as a single serotype although there are two genetically distinct lineages and multiple unique viral clades circulating. When vector-borne flaviviruses are introduced into new systems, there can be a rapid development of viral lineages, genotypes, and serotypes [40,41]. ZIKV is no exception with new genetic changes and syndromic presentations being reported in recent outbreaks [42]. Strain and serotype-specific performance of flaviviruses is well described [29,40,43] and the different inhibition properties of ZIKV shown here and described in the literature may be a function of conformational differences due to changes in amino acid sequences [33,44]. ZIKV MR−766 and other African isolates have been shown to possess significant differences in amino acid sequence from Asian and American isolates [20,21,45]. We were unable to determine whether there might be a potential lineage/serotype behavior present as recent African strains are not readily available for making a reasonable comparison. This issue warrants an in depth investigation especially since the designation of ZIKV as a single serotype was made based on the convalescent serum of two European travelers and mice [46].

The data here indicate that when used for serological diagnostics, ZIKV MR−766 is neutralized in a different manner than ZIKV isolated from the Western Hemisphere, which could lead to the misdiagnosis of subject specimens. Here, most human subjects neutralized MR−766 more effectively than R103451 and PRVABC59. Even in ZIKV-naïve subjects, MR−766 was neutralized better than DENV which could lead to the misdiagnosis of ZIKV when following diagnostic algorithms in pregnant women leading to unnecessary interventions.

The CDC diagnostic criteria for “non-negative” IgM ELISA may not allow for accurate diagnosis of ZIKV or other flaviviruses in areas endemic to multiple flaviviruses. The most recent CDC diagnostic algorithm recommends concurrent testing with DENV to differentiate between ZIKV and other flaviviral infections [47]. However, the CDC does not specify which DENV serotype or whether multiple serotypes should be used [47]. A cursory examination into recent ZIKV serological studies shows that many laboratories employ a single strain of DENV for neutralization assays [48,49,50]. This could also lead to misdiagnosis of ZIKV since data throughout this manuscript have shown that neutralization of DENV by mAbs and subject sera is serotype specific. Perhaps, multiple serotypes of DENV and endemic flaviviruses should be evaluated when endeavoring to diagnose a ZIKV infection. This strategy was employed by Montoya et al in which locally isolated ZIKV and DENV were employed as the medium for their neutralization assays [51]. This group reliably differentiated DENV from ZIKV for all time points evaluated post exposure [51].

Qualitative PRNTs performed at a single serum dilution of 1:10 are commonly used to identify patients with potential exposure in a high throughput manner. The data here show that at the 1:10 dilution, cross-reactive antibodies can identify multiple viruses that would need to be evaluated via a quantitative PRNT. PRNT analysis is time consuming and labor intensive and, given the data here and in the literature, at least five quantitative PRNTs should be performed to verify ZIKV exposure. A better option may lie in performing nucleic acid testing on other types of specimens. The CDC does allow for testing of a variety of biological fluids and even recommends using whole blood in lieu of serum [47,52,53,54].

The use of MR−766 as a diagnostic reagent is of significant concern especially if used in the context of pre-natal care or serosurveillance. The CDC offers ZIKV antigen derived from a variety of ZIKV isolates. BEI Resources and the World Reference Center for Emerging Viruses and Arboviruses (WRCEVA) offer a variety of recent, defined isolates free of charge. While antigens and reagents are commercially available, these products may not disclose the source of antigen.

In areas where flaviviruses co-circulate, subject samples are diagnosed via RT-PCR, IgM ELISA, or by PRNT. Though in most endemic areas, RT-PCR is the most common diagnostic method employed as facilities and expertise are not often available for serological assays. The data here and in the literature show that detection of ZIKV nucleic acids in subject serum is not only time limited, given the short window of viremia, but also strain dependent [2,3,21]. Our data show that when using the CDC diagnostic primer set designed against MR-766, there was reduced detection of nucleic acids from strain PRVABC59. These primers are employed by clinical and research laboratories and are included in the CDC Trioplex real-time RT-PCR assay [25]. Similar findings have been reported by Baylis et al which evaluated the sensitivity of a French Polynesian standard for use in the WHO ZIKV RT-PCR [55,56]. Here, multiple labs reported increased sensitivity for MR−766 and closely related French Polynesian isolates that contain the A266V mutation but reduced sensitivity for Cambodian and Puerto Rican isolates that lack the mutation [56].

RT-PCR and antigen preparations using locally circulating strains should be employed in areas of cocirculating arboviruses. Strain-dependent RT-PCR sensitivity should be recognized as a potential means for no or equivocal detection of ZIKV in blood bank and clinical specimens collected prior to IgM production.

## 4. Methods

### 4.1. Viruses

All experiments were performed using the initial expansion of virus from Vero cells. Viruses were obtained from BEI resources and WRCEVA. ZIKV PRVABC59 (Cat. # NR−50240 Lot# 64112564), was isolated from a human subject in Puerto Rico during 2015 and is of Asian lineage. ZIKV R103451 (Cat. # NR−50355 Lot# 64362036) is also of Asian lineage and was isolated from a human placenta during 2016 from a subject who had traveled to Honduras the previous year. ZIKV MR−766 (Cat. # NR−50065) is a prototype isolate of African lineage that was isolated from a rhesus monkey in the Zika forest of Uganda during 1947 [6].

A DENV 1−4 diagnostic reference panel was obtained from BEI Resources and included: DENV1 TS-SMAN (Cat #: NR−83 Lot# 57982370), DENV2 New Guinea C (WRCEVA, TVP 10815), DENV3 Philippines/H87/1956 (WRCEVA, TVP 15322), and DENV4 H241 (WRCEVA, TVP 13886). Other reference flaviviruses were obtained from the World Reference Center for Emerging Viruses and Arboviruses and included: Japanese Encephalitis virus SA−14−14−2 (WRCEVA, TVP 23110), and Yellow Fever virus 17D (Cat# NR−115 Lot# 7496108). West Nile virus (WNV) strain New York 99 was kindly provided by Dr. Long and was from the second passage of virus isolated from a crow during the 1999 WNV outbreak in New York. Aside from WNV, all viruses used in this study were from stocks which were expanded once upon receipt from provider in Vero E6 cells, titrated in triplicate via viral plaque assay in Vero E6 cells and then stored at −80 °C. Titer was calculated as plaque forming units per mL.

### 4.2. Monoclonal Antibodies

Three different mAbs were chosen for this project based on their availability, transparent history and production, and use in other scientific publications (Table 3). The 4G2 antibody was purified from D1−4G2−4−15 hybridoma cells (ATCC #HB−112) cultured in RPMI medium with 10% FBS. This mAb targets a highly conserved portion of the Domain 3 flavivirus envelope glycoprotein and has been shown to react with most flaviviruses [36]. The mAb ZKA185 was produced from B cells derived from ZIKV-infected but DENV naïve donor [33]. This antibody is reported to neutralize ZIKV but not DENV [33]. The mAb 753(3) C10 binds to the envelope dimer region and was produced from a human subject hospitalized with DENV hemorrhagic fever with RT-PCR-confirmed infection with DENV1 [35]. Both 753(3) C10 and ZKA185 were obtained commercially (Absolute Antibody, Oxford, UK).

### 4.3. Subject Specimens

ZIKV-naïve clinical human specimens with flaviviral exposure history were obtained through an ongoing study enrolling subjects with symptoms of arboviral disease in Pakistan [8,59]. Specimens were collected at presentation to health care with symptoms of acute febrile illness. Informed consent and study procedures were reviewed and approved by the Ethics Review Committee at Aga Khan University (#3183-PAT-ERC−14). All enrolled subjects gave written consent in accordance with the Declaration of Helsinki. Subjects were not vaccinated for JEV or YFV. De-identified, curated human specimens of verified ZIKV exposure but unknown flavivirus exposure were obtained from BEI Resources. Serum from 4 adult female specific pathogen-free Polypay sheep (New England Ovis) was included as a flavivirus naïve background. These sheep were part of a separate ongoing study to evaluate the consequences of congenital ZIKV infection. Sheep were infected with 10^8^ infectious units of ZIKV R103451 intravenously. Specimens for this study were collected 4 weeks post inoculation following guidelines approved by the University of Florida IACUC protocol #201609345. Sheep were housed under BSL2+ containment and husbandry conditions.

### 4.4. Serologic and Molecular Assays

PRNTs of subject samples were performed on confluent monolayers of Vero E6 cells. 100 infectious units (i.u.) of virus (calculated from stock virus titer) in PBS were incubated for 1 hour at 37 °C with 4-fold serial dilutions of subject serum. Assay controls included 100 i.u. of virus in PBS, 100 i.u. of virus with 1:10 dilution of positive control serum, and a mock infected control consisting of PBS. Cells with inoculum were incubated at 37 °C for one hour, after which the inoculum was removed and an overlay consisting of MEM with 10% FBS and 0.5% methylcellulose. Assays were incubated 3−7 days, depending on virus, at 37 °C after which monolayers were stained with Coomassie blue.

RT-PCR for ZIKV isolates was performed using the CDC diagnostic one-step RT-PCR protocol with ZIKV general primers and probe [2,6]. These primers are also found in the United States Food and Drug Administration FDA-approved CDC Trioplex real-time PCR assay from CDC [25,26].

Virus inhibition assays using monoclonal antibodies were performed using serial dilutions of mAb diluted in PBS as described elsewhere [60,61]. Briefly, 100 i.u. of virus (as determined by viral plaque titration) were incubated with mAb in PBS for 1 hour at 37 °C after which Vero cells were inoculated with the mixture and incubated for 1 hour at 37 °C. The inoculum was then removed, and the cells rinsed with PBS to remove any residual mAb and then the cells were covered with a 0.5% methylcellulose overlay and allowed to incubate at 37 °C until viral plaques formed (3−7 days depending on virus). Results are expressed as the average of at least two independent trials with 2 technical replicates for each dilution. Assay controls included 2 wells each for mock infection, virus only, and mAb with a known neutralized virus. Percent neutralization, inhibition, and enhancement were calculated using the virus only well as the baseline value.

### 4.5. Statistical Analysis

Subject data were analyzed using MedCalc 64 Bit statistical analysis software. Non-linear regression analysis was performed for all inhibition and neutralization assays. Interpolation of the data was fit with a polynomial curve. Multiple regression analysis was performed to identify statistical significance between the virus strains. RT-PCR data were analyzed using the ΔΔCt method to calculate fold change in detection of the viruses. Pairwise comparisons of Ct values were performed using Student’s T-test. The virus only control was used as the reference sample for all analyses.

## 5. Conclusions

When exposure history is unknown, ZIKV diagnostics are complicated and ZIKV MR−766 should not be used as a reagent. Serologic diagnosis of ZIKV, or any other flaviviruses, cannot be consistently or reliably achieved under CDC guidelines, where arboviruses co-circulate and may not be appropriate in these areas due to serologic cross-reactivity. Unfortunately, we were unable to obtain an appropriate number of known, flaviviral-infected human specimens to fully investigate this issue of flaviviral cross-reactivity. Even the CDC had very few known human specimens for evaluating cross-reactivity of their MAC ELISA [9]. In this manuscript, we have explored the influence of viral strain and subject specimens on ZIKV serological diagnostics. Our data show that further investigation of flaviviral cross-reactivity is needed. Perhaps suspect patients should be assessed more frequently by RT-PCR of whole blood in ZIKV endemic regions. While ZIKV was the focus of this paper, it deserves mention that neutralization of YFV and JEV was observed in subjects inhabiting non-endemic regions and without vaccination history. The inconsistent activity of individual ZIKV isolates highlights the necessity of characterizing virus and reagents for diagnostic use.

## Figures and Tables

**Table 1:** Differential RT-PCR detection of three Zika virus (ZIKV) strains. Three ZIKV strains detected with CDC ZIKV general primers and probe. MR−766 was used as the reference when calculating Ct, fold change, and *p*-values.

	AVG Ct ± SE	AVG CT ± SE	AVG Fold Change ± SE	Log Fold Change	*p*-Value
MR−766	21.6 ± 0.04				
PRVABC59	25.18 ± 0.02	3.57 ± 0.01	0.08 ± 0	−3.5767	0.0000014
R103451	21.02 ± 0.06	−0.58 ± 0.03	1.5 ± 0.03	0.5863	0.0035

**Table 2:** Cross neutralization of flaviviruses by subjects with and without ZIKV exposure. Data denotes average neutralization ± standard deviation.

Patient ID	Virus						
	DENV 1	DENV 2	DENV 3	DENV 4	YFV	WNV	JEV
**ZIKV Exposure with Unknown Exposure History**							
50616	0 ± 0	0 ± 0	0 ± 0	0 ± 0	0 ± 0	0 ± 0	0 ± 0
50620	0 ± 0	0 ± 0	99.5 ± 0.7	0 ± 0	0 ± 0	0 ± 0	67 ± 0.7
50622	0 ± 0	0 ± 0	99.5 ± 0.7	0 ± 0	97 ± 0.7	0 ± 0	61 ± 2.1
125	0 ± 0	0 ± 0	0 ± 0	0 ± 0	0 ± 0	0 ± 0	0 ± 0
88	0 ± 0	100 ± 0	99.5 ± 0.7	0 ± 0	92 ± 1.4	0 ± 0	13 ± 3.5
**Flavivirus Naïve SPF Sheep with ZIKV Infection**							
4155	0 ± 0	100 ± 0	0 ± 0	0 ± 0	0 ± 0	0 ± 0	79 ± 0.7
4158	0 ± 0	0 ± 0	0 ± 0	0 ± 0	97 ± 0.7	0 ± 0	78 ± 5.6
4072	0 ± 0	78 ± 1.4	0 ± 0	0 ± 0	0 ± 0	0 ± 0	82 ± 0
4171	0 ± 0	0 ± 0	0 ± 0	0 ± 0	100 ± 0	0 ± 0	0 ± 0
**ZIKV Naïve with Unknown Flavivirus Exposure**							
315	100 ± 0	0 ± 0	100 ± 0	0 ± 0	86.6 ± 1.4	85.3 ± 4.2	93.2 ± 2.8
070	100 ± 0	100 ± 0	0 ± 0	0 ± 0	96.6 ± 1.4	47 ± 9.9	0 ± 0
200	100 ± 0	100 ± 0	100 ± 0	100 ± 0	55 ± 6.3	0 ± 0	92.2
252	98.1 ± 2.5	100 ± 0	100 ± 0	100 ± 0	0 ± 0	58.5 ± 1.4	91.2

**Table 3:** Monoclonal antibodies used in this study.

mAb	Source	Neutralization	Target	Reference
4G2	mouse	Dengue virus (DENV) and other flaviviruses	DENV fusion loop—Envelope domain 2	[36,57]
ZKA185	human	ZIKV	ZIKV Envelope Domain 2	[33,58]
753(3) C10	human	ZIKV, DENV	Envelope dimer region	[35]
